# Investigating the relationships between motor skills, cognitive status, and area deprivation index in Arizona: a pilot study

**DOI:** 10.3389/fpubh.2024.1385435

**Published:** 2024-06-25

**Authors:** Madeline Hooten, Marcus Ortega, Adewale Oyeyemi, Fang Yu, Edward Ofori

**Affiliations:** ^1^College of Health Solutions, Arizona State University, Phoenix, AZ, United States; ^2^Edson College of Nursing and Healthcare Innovation, Arizona State University, Phoenix, AZ, United States

**Keywords:** cognitive status, social determinants of health, motor reserve, brain health and performance, middle aged adults, area deprivation index (ADI)

## Abstract

**Introduction:**

Previous studies highlight the negative impact of adverse socioeconomic conditions throughout life on motor skills and cognitive health. Factors such as cognitive activity, physical activity, lifestyle, and socioeconomic position significantly affect general health status and brain health. This pilot study investigates the relationships among the Area Deprivation Index (ADI)—a measure of neighborhood-level socioeconomic deprivation, brain structure (cortical volume and thickness), and cognitive status in adults in Arizona. Identifying measures sensitive to ADI could elucidate mechanisms driving cognitive decline.

**Methods:**

The study included 22 adults(mean age = 56.2 ± 15.2) in Arizona, residing in the area for over 10 years(mean = 42.7 ± 15.8). We assessed specific cognitive domains using the NeuroTrax™ cognitive screening test, which evaluates memory, executive function, visual–spatial processing, attention, information processing speed, and motor function. We also measured cortical thickness and volume in 10 cortical regions using FreeSurfer 7.2. Linear regression tests were conducted to examine the relationships between ADI metrics, cognitive status, and brain health measures.

**Results:**

Results indicated a significant inverse relationship between ADI metrics and memory scores, explaining 25% of the variance. Both national and state ADI metrics negatively correlated with motor skills and global cognition (*r*’s < −0.40, *p*’s < 0.05). In contrast, ADI metrics generally positively correlated with motor-related volumetric and cortical thickness measures (*r*’s > 0.40, *p*’s < 0.05).

**Conclusion:**

The findings suggest that neighborhood-level social deprivation might influence memory and motor status, primarily through its impact on motor brain health.

## Introduction

The World Health Organization (WHO) has highlighted the significance of non-communicable diseases (NCDs) and their impact on global health, underscoring the importance of addressing risk factors across all age groups to prevent disability and enhance quality of life (1). Motor skills, encompassing both fine and gross motor abilities, are crucial for daily functioning and independence among middle-aged adults. Fine motor skills may become compromised due to decreases in motor capacity in older adults, however, this decline can be influenced by a myriad of factors including biological, lifestyle, physiological and environmental conditions (2–7). Further, maintaining brain health is integral to preserving motor skills, which is vital for preventing disability and promoting quality of life, thereby directly addressing one of the key risk factors for NCDs across all age groups.

Socioeconomic factors, such as education and financial stability, play a pivotal role in the development and maintenance of motor skills. Research has demonstrated that lower socioeconomic status is associated with poorer health outcomes, including a decline in motor skills (8–12). This decline not only affects an individual’s ability to perform daily activities but also increases their dependency, thereby impacting their quality of life and imposing economic burdens on healthcare systems and caregivers. Furthermore, 1 in 11 people aged 45 years and older have reported experience subjective cognitive decline (SCD) and the state of Arizona had the 7th highest prevalence of individuals reporting SCD (13). SCD is usually associated with functional impairment with greater than 50% reporting challenges (13).

The social determinants of health (SDOH) are integral in understanding the environmental and societal factors that contribute to health disparities. The Area Deprivation Index (ADI) is a novel tool that reflects neighborhood-level socioeconomic disadvantages, encompassing 17 dimensions of SDOH based on data from the American Community Survey and the United States Census Survey (3). Studies utilizing ADI have shown that living in socioeconomically disadvantaged neighborhoods is linked to adverse health outcomes, including chronic conditions, cognitive impairment and reduced motor skills (3, 7, 14). It is currently unknown how ADI affects memory and motor skills in Arizona.

Life course theory suggests that exposure to various biological, psychological, environmental, and lifestyle factors from an early age influences health trajectory (15, 16). Further, participations in varying mobility activities may influence specific physical-motor development and later life trajectories (17, 18). This theory underscores the importance of considering long-term exposure to socioeconomic disadvantage and its cumulative effects on health, including motor skills (19). Previous reports that indicate extracurricular physical activities can influence motor skills and cognition and provide a potential modifiable factor for SCD (20, 21). Higher ADI values have been associated with faster cognitive decline in older adults; however, the relationship between ADI and motor skill decline in older adults requires further investigation to develop targeted interventions (7, 14).

The inconsistent relationships between neighborhood socioeconomic disadvantage and health outcomes, including motor skills, highlight the need for a better understanding of how individual and neighborhood-level socioeconomic factors interact to influence health (22). By extending this framework to motor skills in middle-aged adults, research can illuminate pathways through which socioeconomic and environmental conditions impact physical functioning and independence, informing policies and interventions aimed at improving global health outcomes in this demographic.

The purpose of the manuscript is to examine whether ADI impacts cognitive and motor status in adults in Arizona. We hypothesize ADI would be related to memory and executive function. We also want to explore whether ADI metrics also are associated with brain health measures of volume and cortical thickness.

## Methods

### Participants

To be included in this study, all participants had to reside in the state of Arizona and attend one in-person testing session at the Arizona State University Downtown Phoenix campus. Participants had to be 18 years or older with no significant cognitive impairment during their cognitive testing session. Participants were excluded if they had a history of stroke, brain ischemia, or any other contraindications for MRI. Participants’ MRI scans were collected at Banner Alzheimer’s Institute (BAI) before NeuroTrax was administered on-site on participants’ designated testing day. The Rapid Assessment of Physical Activity (RAPA) questionnaire was administered via REDCap adaptation and electronically sent to participants before their on-site testing session. Data was collected through the use of the ADI dashboard and REDCap. A current address was provided for all 22 participants and inputted in the Neighborhood Atlas to generate a state rank and a national percentile. NeuroTrax data was obtained onsite via a laptop, mouse, and numeric keypad. Participants were set up in a quiet room with minimal distractions and asked to work through the computerized battery until completion. Participants on average took 45 min to an hour to complete the computerized assessment. Participants completed these surveys via a REDCap invitation sent to their email, which notified staff upon completion. A report was generated through the data export tool in REDCap for further analysis post-collection.

### Area deprivation index

National and state ADI data was calculated using the Neighborhood Atlas^®^ by the Health Resources & Services Administration (HRSA), from the University of Wisconsin-Madison School of Medicine and Public Health (5). Participant addresses were inputted into the Neighborhood Atlas to produce a state rank according to their residential census block group. Ranks were distributed on a scale of 1 through 10, with 10 representing the most disadvantaged block groups (5). The national percentage was obtained through the same ranking process and ranked participants into percentiles in increments of ten starting from one to 100. Most disadvantaged groups were ranked in the highest percentile (5).

### Brain health

Brain Health was assessed with FreeSurfer 7.2 (23) by calculating structural and cortical thickness measurements. Briefly, the protocol includes automated spatial normalization, intensity normalization, non-brain tissue removal, and segmentation of cortical and subcortical structures for each hemisphere. This process facilitates detailed measurements of cortical thickness and volume. We utilized regional and intracranial volume (ICV) correction to account for various head sizes. Selected regions of interest for memory-related regions were entorhinal cortex, fusiform gyrus, parahippocampal gyrus, precuneus, and anterior cingulate cortex (24–26). Regions of interest for motor skill-related regions were paracentral lobule, precentral gyrus, caudal middle frontal gyrus, and postcentral gyrus (27).

### Cognitive status

NeuroTrax™ is a series of Mindstream tests used to generate a global score inclusive of cognitive domain measures (memory, executive, visuospatial, verbal, attention, and information processing speed) (22, 28, 29). For the purposes of this study, cognitive function was evaluated using the validated computerized NeuroTrax cognitive screening test to obtain cognitive domain measures of executive function (EF), attention, memory, visual–spatial processing (VSP), verbal function, motor function, information processing speed (IPS), and a global cognitive score (GCS). Normative data regarding all motor skill and cognitive tests can be found at: https://portal.neurotrax.com/docs/norms_guide.pdf.

### Motor skills status

The motor skill assessments consisted of finger tapping and a catch game designed to evaluate fine motor skills. For the finger tapping task, participants were presented with a white rectangle that gradually filled with red from left to right over 12 s. They were instructed to tap the mouse with their finger as many times as possible during this period. The research staff recorded the responses using a mouse and a number keypad, with outcome measures being the intertap interval and associated variance in milliseconds.

The second test was the “catch” game, which assessed the preparation and execution of movements through an engaging video game format. This computerized system utilized adaptive testing and precise timing. During the game, participants saw a rectangular white object falling vertically from the top of the screen. Their task was to position a rectangular green paddle directly in the path of the falling object before it reached the bottom of the screen. The paddle could be moved horizontally across the bottom of the screen by pressing the left mouse button to move it leftward and the right button to move it rightward. Participants used their best hand for responses.

As the test progressed, the rate of the falling object increased incrementally, making it increasingly difficult to “catch” the object in time. Outcome parameters included response time and associated variance for the first move, the number of direction changes per trial, errors for missed catches, and a total performance score. After each battery was completed, the research staff received a detailed report of all cognitive domain areas, which they utilized for further analysis.

### Rapid assessment of physical activity

Participant’s physical activity level was measured via REDCap adaptation of the Rapid Assessment of Physical Activity (RAPA). RAPA is a validated physical activity questionnaire primarily used by clinicians to assess individual physical activity levels in older adults (30). RAPA is a nine-item questionnaire with response options of “yes” or “no” to questions related to physical activity (31). The total score of the first seven items ranges from one to seven points, with the respondent’s score categorized into one of five levels of physical activity; 1 = sedentary, 2 = underactive, 3 = regular underactive (light activities), 4 = regular underactive, and 5 = regular active. Responses to the strength and flexibility items were scored separately; strength = 1, flexibility = 2, or both = 3 (31). Physical activity was assessed to understand possible interactions between brain volume and ADI.

### Statistical analysis

Data analysis was conducted using IBM SPSS Statistics version 29 (IBM Corp., Armonk, NY). The analysis involved several steps to thoroughly examine the relationships between various metrics and outcomes. Descriptive statistics were calculated for all variables, including means, standard deviations, and percentiles, to provide an over for the relevant variable. Linear regression analyses were conducted to assess the associations between Alzheimer’s Disease Index (ADI) metrics (i.e., State and National percentiles) and dependent variables such as cognitive status, motor skill status, and brain health regions. The dependent variables included NeuroTrax cognitive domain scores, motor skill scores, and brain health measures obtained from neuroimaging data. Beta weights (β) or standardized coefficients were reported for each predictor variable to indicate the strength and direction of the association. Statistical significance was determined at the *p* < 0.05 level. Assumptions of linear regression were checked, including linearity, normality of residuals, homoscedasticity, and absence of multicollinearity (Supplementary Table S8). Diagnostic plots (e.g., residual plots, Q-Q plots) were examined to ensure the validity of the regression models (see Supplementary Figure S2). The Durbin-Watson test and Runs test were used to check for independence of residuals. The Shapiro–Wilk test was used to assess the normality of residuals. The Condition Index was used to detect multicollinearity. Pearson correlation coefficients were calculated to explore the relationships among brain health regions, the Rapid Assessment of Physical Activity (RAPA) scores, and NeuroTrax cognitive scores. This analysis provided insights into the relationships between physical activity, cognitive performance, and specific brain health metrics. Correlation coefficients (r) were reported, indicating the strength and direction of the linear relationship between pairs of variables. The significance of the correlations was evaluated at the *p* < 0.05 level. All analyses were conducted with a significance level set at *p* < 0.05.

## Results

The demographic and clinical characteristics of the sample, comprising an average age of 53.7 years (SD = 19.2), revealed a predominance of female participants (81.8%) and non-Hispanic whites (86.5%) (See Table 1). Participants reported an average of 17.8 years of education (SD = 2.9) and approximately 7 h of sleep in the last 24 h (SD = 1.2). Physical activity, as assessed by the RAPA Scale, yielded a mean score of 2.5 (SD = 1.2). The NeuroTrax Global Score averaged at 104.1, indicating the overall cognitive function of the cohort. Socioeconomic status, gauged through ADI, positioned the sample at the 34th national percentile and within the 3rd state decile (SD = 1.6), suggesting moderate socioeconomic challenges.

**Table 1:** Sociodemographic characteristics.

	Sample
	Number or Mean (SD)
Age (Mean, SD)	53.7 (19.2)
Sex (F,%)	81.8%
Ethnicity (non-Hispanic %)	86.4%
Years of Education (13 or more years in school)	17.8 (2.9)
Years lived in Arizona	42.7 (15.9)
Hours of sleep in the last 24 h (Mean, sd)	6.7 (1.2)
Rapid Physical Activity Scale 1	2.5 (1.2)
NeuroTrax Global Score	99 (12.3)
Area Deprivation Index, National Percentile	37th (16)
Area Deprivation Index, State Decile	3rd (1.9)

SD, standard deviation.

For means and standard errors of NeuroTrax (i.e., cognitive domain metrics) and brain health measures (see Supplementary Tables S1–S3). Linear regression analyses delineated relationships between cognitive domains and socioeconomic status. Memory subscale scores were inversely associated with state decile levels (*p* < 0.05, see Table 2). The NeuroTrax Global Score’s association with the ADI, at both the national percentile (*r* = −0.46, *p* < 0.05) and state decile levels (*r* = −0.43, *p* < 0.05), demonstrated a strong negative correlation. Further, motor skills exhibited moderate negative associations with national (*r* = −0.52, *p* < 0.05) and state (*r* = −0.48, *p* < 0.05) ADI levels.

**Table 2:** Linear regression analyses between NeuroTrax and area deprivation indices.

	National percentile			State Decile		
	β	*t*	*p*-value	β	*t*	*p*-value
Global score	**−0.46**	**−2.23**	0.038*	**−0.43**	**−2.1**	0.049*
Memory	−0.31	−1.40	0.178	**−0.49**	**−2.5**	0.023*
Executive function	−0.12	−0.52	0.608	−0.33	−1.5	0.147
Attention	−0.28	−1.25	0.225	−0.40	−1.9	0.070
Information processing	−0.20	−0.90	0.382	−0.32	−1.5	0.158
Visual spatial skills	−0.38	−1.76	0.094	−0.10	−0.5	0.654
Motor skills	**−0.52**	**−2.63**	0.017*	**−0.48**	**−2.36**	0.029*

*Notes significance at the 0.05 level. Bold values are intended to indicate parameters that met significance.

**Figure 1:**
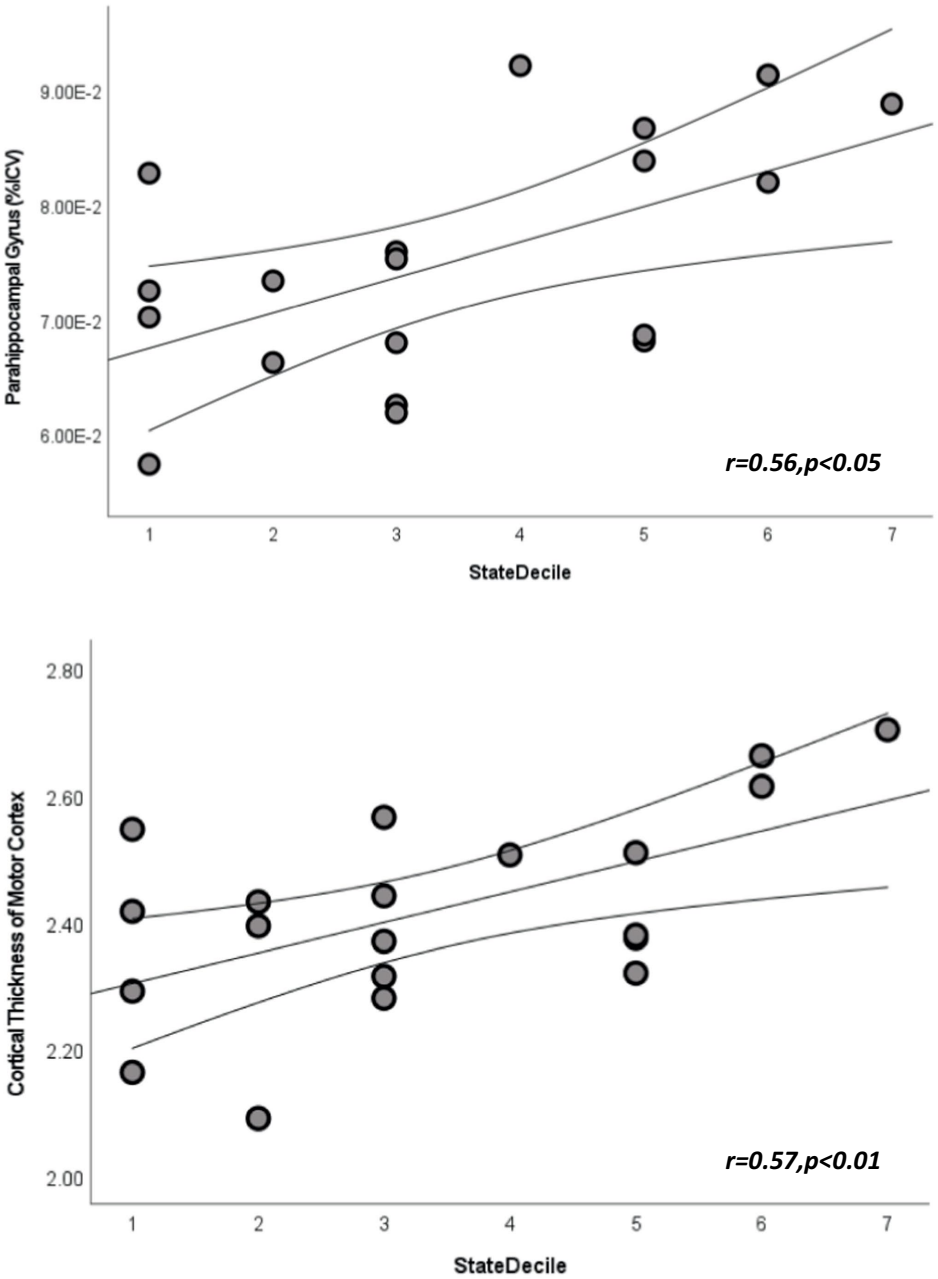
Scatterplots depicting the relationship between state area deprivation indices and cortical volume from parahippocampal gyrus (top) and cortical thickness from paracentral lobule (bottom). Straight lines represent best fit line for each plot and curved lines represent 95% confidence intervals.

Linear regression analyses also revealed significant relationships with cortical thickness and intracranial volume measures (*p*’s < 0.05, see Figure 1; Supplementary Tables S4, S5). More significant regions were found among motor-related regions, state and national ADI (*p* < 0.05, see Figure 1; Supplementary Tables S4, S5). The only memory-related region that revealed significance was precuneus regional volume with state decile ADI metrics (*r* = 0.61, *p* < 0.01).

Given the unexpected direction of findings we decided to explore the Pearson correlations among NeuroTrax scores and brain health regions to determine whether our findings could be hypothesis generating or perhaps incidental (see Supplementary Figure S1; Supplementary Tables S6, S7). We can see that in the current cohort that mostly default mode and memory network regions had the largest associations with NeuroTrax scores, which are expected, with entorhinal cortical thickness revealed the largest association (*r* = 0.62, *p* < 0.05) with memory. Further, RAPA scores were inversely related with motor areas (see Supplementary Figure S1).

## Discussion

In our exploratory study, we investigated the potential relationship among memory, motor skills, and global cognition and socioeconomic status, as measured by ADI, within a small cohort of mostly middle-aged individuals from Arizona. Our findings suggest that ADI may influence cognitive status and that this relation may be potentially compensated through motor-related brain regions. This tentative observation suggests that environmental and socioeconomic conditions could influence cognition, potentially compensated by changes in motor-related brain regions.

### NeuroTrax scores and ADI

Research on motor skills has predominantly focused on children, examining the development and refinement of these skills from early childhood (32, 33). Our study probes the connection between socioeconomic factors, measured through ADI and cognitive status among middle-aged adults living in AZ. Our approach highlights the importance of considering how environmental factors continue to influence physical abilities well beyond childhood and adolescence. Our cohort, having resided in Arizona for most of their lives, potentially strengthens the evidence of the long-term distal effects that ADI can have on cognition and motor status throughout the lifespan. Our findings extend findings suggesting that ADI is associated with memory and extends this to motor status (34, 35). The link between cognitive function and socioeconomic status is well-documented, with studies indicating that individuals from socioeconomically disadvantaged backgrounds tend to exhibit lower cognitive performance. Albeit, this may be due to a variety of factors such as cultural appropriate testing, biases in ethnoracial sampling, access to care, and lack of performance match scoring studies. The current research suggests local metrics of ADI influence motor brain health underscores the impact of environmental stressors, educational access, and healthcare availability on cognitive health (36).

Our novel results suggest that ADI influences motor skills in adults, suggesting that socioeconomic conditions may similarly affect physical health, albeit these findings are preliminary and necessitate further investigation due to the limited scale of our research. The literature regarding motor skill competence linked to SES metrics are mixed (37–39). Our findings are in line with literature suggesting increases in SES lead to worse performance and extend to older adults as most literature report effects in toddlers and adolescents (40, 41). Further our results suggest that mechanism by which ADI impacts cognition and motor function may be similar. We also propose that a common adaptive link may underlie motor brain health and ADI (42, 43).

The underlying mechanisms that might explain our observed association between motor skills and ADI are likely multifaceted, encompassing environmental, psychological, and physiological elements (44). Socioeconomic disadvantage is often synonymous with increased environmental pollutants, reduced access to physical activity facilities, and elevated stress levels, all of which could potentially impair motor skill acquisition, cognition and maintenance. Moreover, disparities in educational and recreational opportunities could further compound these effects. Our findings are in line with literature suggesting psychomotor tests to reflect sociocultural characteristics (39).

### Brain health metrics and ADI

The interplay between social determinants of health and brain morphology has long been discussed (3, 45, 46). Our study reveals an interesting pattern: motor-related areas of the cortex show notable increases in volume and thickness with increases in socioeconomic deprivation. This pattern is especially evident at the state level, suggesting that the environmental stressors associated with deprivation might trigger compensatory neural plasticity or alternative neurodevelopmental paths (47). Key areas in the motor network—such as the precentral gyrus, paracentral lobule, and postcentral gyrus—exhibit enhanced structural integrity in contexts of socioeconomic disadvantage, possibly reflecting the brain’s adaptive response to more challenging physical and psychological environments (48). Our findings indicating structural associations with increases with ADI are similar to studies suggesting compensatory processing or increased activation is necessary for improved performance (49, 50). Given that on average, there were no deficits indicated globally as participants scored within normal range on the test further strengthens this possibility potential in one’s environment. The cortex’s inherent plasticity and its role in behaviors highly dependent on environmental factors might make it particularly vulnerable to modifications induced by socioeconomic adversity. This distinction invites deep inquiry into the mechanisms driving these region-specific socioeconomic effects and their potential consequences. Are these changes a positive adaptation or a detrimental result of prolonged stress? Unraveling these complexities is crucial for observing modifiable behaviors aimed at mitigating the negative effects of socioeconomic disparities on brain development and performance. To our knowledge this is the first study to determine how ADI impacts motor function in older adults.

Despite the inherent limitations of our study, including its small sample size, it underscores the critical need to consider a wide array of factors, such as physical activity and mental health, in understanding the multifaceted relationship between socioeconomic status and health outcomes. Physical activity, for instance, might offer a mitigating effect against the negative impacts of socioeconomic disadvantage on health, whereas mental health challenges like depression could exacerbate them (51). Further other limitations that may warrant further study are BMI, social support and family structure, financial stress, medical and lifestyle factors that also may impact deprivation indices (52–54).

In conclusion, our pilot study contributes to the dearth body of literature exploring the effects of socioeconomic factors on motor skills in middle-aged populations. While our findings are preliminary and necessitate cautious interpretation, they pave the way for future research to more definitively explore these relationships. Addressing socioeconomic disparities to improve health outcomes remains a critical area for further investigation, emphasizing the need for comprehensive interventions that target both cognitive and physical health across the lifespan.

## Data availability statement

The raw data supporting the conclusions of this article will be made available by the authors, without undue reservation.

## Ethics statement

The studies involving humans were approved by Arizona State University IRB Board. The studies were conducted in accordance with the local legislation and institutional requirements. The participants provided their written informed consent to participate in this study.

## Author contributions

MH: Data curation, Project administration, Supervision, Writing – original draft, Writing – review & editing. MO: Data curation, Formal analysis, Software, Writing – review & editing. AO: Writing – review & editing. FY: Writing – review & editing. EO: Conceptualization, Formal analysis, Methodology, Resources, Software, Supervision, Visualization, Writing – original draft, Writing – review & editing.
