# Supplementary material for: Investigating the relationships between motor skills, cognitive status, and area deprivation index in Arizona: a pilot study
Source: Front Public Health. 2024 Jun 25;12:1385435. doi: 10.3389/fpubh.2024.1385435 (PMC11231207; doi:10.3389/fpubh.2024.1385435)
Supplement: Supplementary file 1 [file Data_Sheet_1.docx]

Supplementary Figure 1.


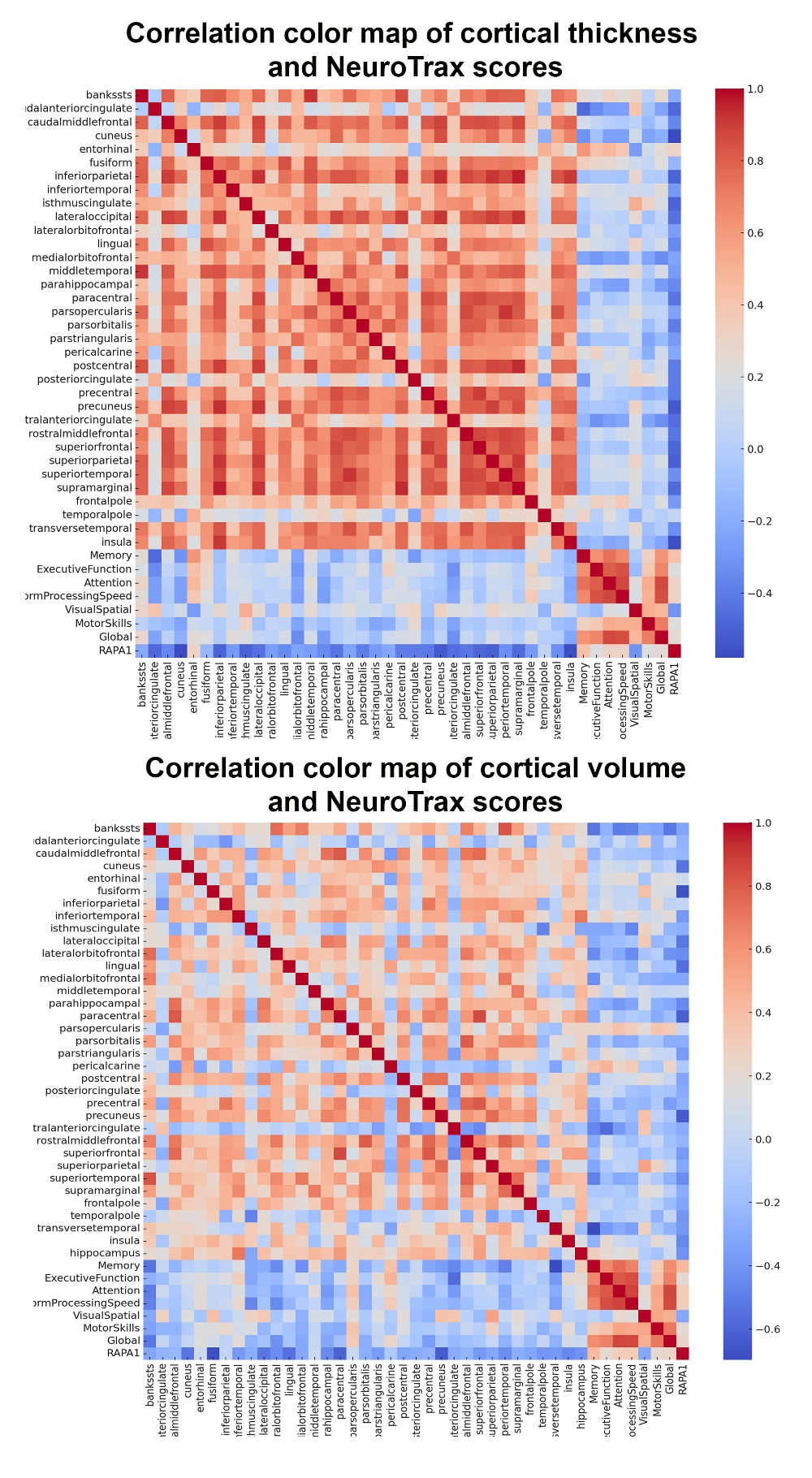


Supplementary Figure 2. Diagnostic Plots of Significant Linear Regression Findings


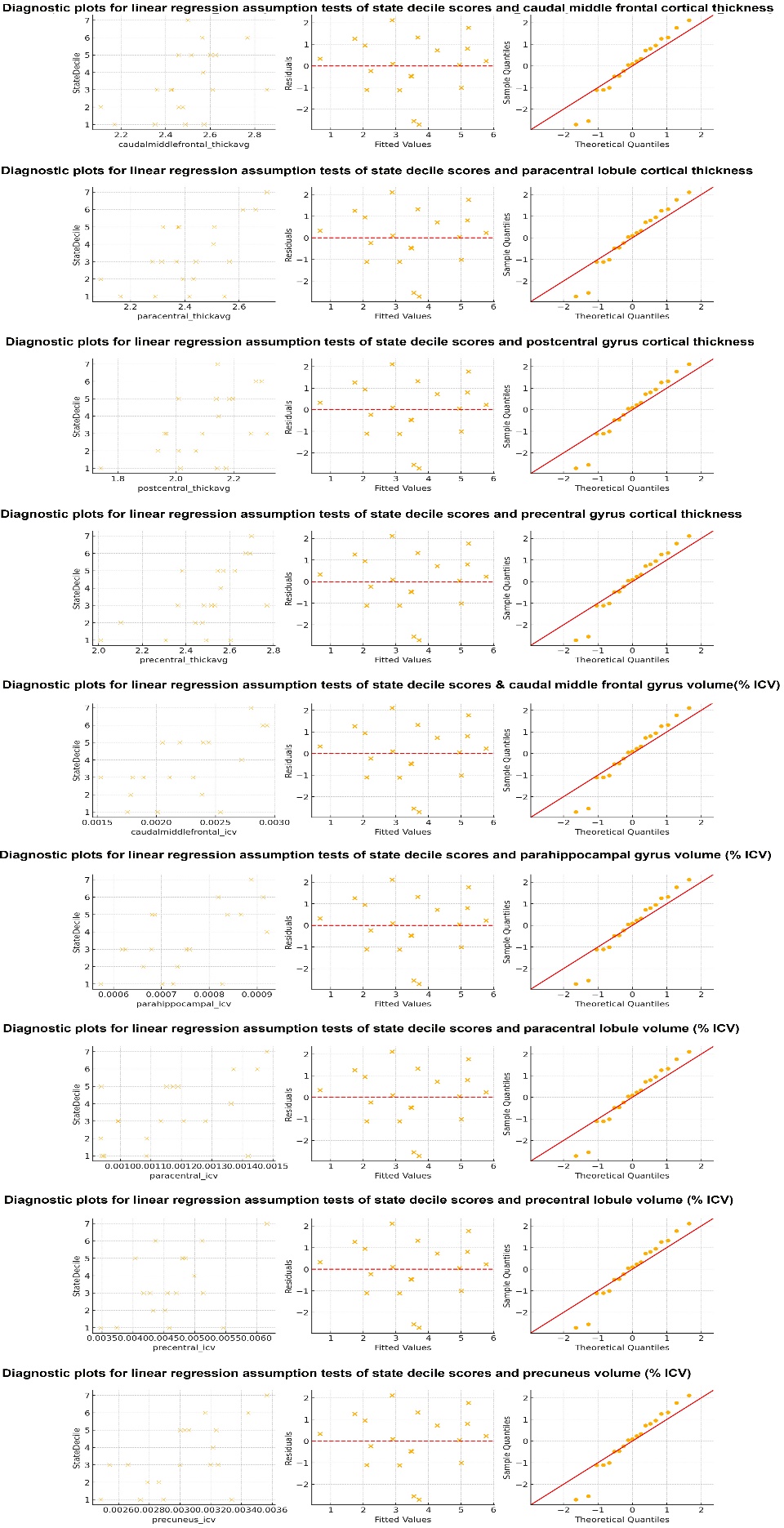


Supplementary Table 1. Mean and standard error (S.E.) of NeuroTrax Scores

| NeuroTrax Variable | Mean | S.E. |
| --- | --- | --- |
| Memory | 100.2 | 3.3 |
| Executive Function | 103.5 | 2.2 |
| Attention | 99.4 | 2.6 |
| Information Processing Speed | 101.3 | 3.9 |
| Visual Spatial | 104.0 | 2.9 |
| Motor Skills | 97.2 | 2.1 |

Supplementary Table 2. Mean and standard error (S.E.) of cortex volumetric measures as a percent of total intracranial volume

| Cortical variable | Mean | Standard Error |
| --- | --- | --- |
| bankssts | 0.074% | 0.0034% |
| caudalanteriorcingulate | 0.058% | 0.0028% |
| caudalmiddlefrontal | 0.224% | 0.0095% |
| cuneus | 0.106% | 0.0032% |
| entorhinal | 0.073% | 0.0035% |
| fusiform | 0.314% | 0.0109% |
| inferiorparietal | 0.396% | 0.0117% |
| inferiortemporal | 0.382% | 0.0093% |
| isthmuscingulate | 0.083% | 0.0019% |
| lateraloccipital | 0.390% | 0.0098% |
| lateralorbitofrontal | 0.265% | 0.0047% |
| lingual | 0.220% | 0.0051% |
| medialorbitofrontal | 0.183% | 0.0038% |
| middletemporal | 0.352% | 0.0098% |
| parahippocampal | 0.075% | 0.0024% |
| paracentral | 0.116% | 0.0042% |
| parsopercularis | 0.173% | 0.0053% |
| parsorbitalis | 0.077% | 0.0024% |
| parstriangularis | 0.131% | 0.0040% |
| pericalcarine | 0.076% | 0.0032% |
| postcentral | 0.326% | 0.0088% |
| posteriorcingulate | 0.098% | 0.0026% |
| precentral | 0.463% | 0.0141% |
| precuneus | 0.302% | 0.0068% |
| rostralanteriorcingulate | 0.087% | 0.0024% |
| rostralmiddlefrontal | 0.508% | 0.0107% |
| superiorfrontal | 0.752% | 0.0210% |
| superiorparietal | 0.432% | 0.0141% |
| superiortemporal | 0.415% | 0.0084% |
| supramarginal | 0.354% | 0.0113% |
| frontalpole | 0.032% | 0.0012% |
| temporalpole | 0.098% | 0.0035% |
| transversetemporal | 0.040% | 0.0015% |
| insula | 0.241% | 0.0058% |

Supplementary Table 3. Mean and standard error (S.E.) of cortical thickness measures

| Cortical Variable | Mean | SE |
| --- | --- | --- |
| bankssts | 2.38 | 0.035 |
| caudalanteriorcingulate | 2.50 | 0.037 |
| caudalmiddlefrontal | 2.50 | 0.039 |
| cuneus | 1.98 | 0.019 |
| entorhinal | 3.20 | 0.061 |
| fusiform | 2.67 | 0.031 |
| inferiorparietal | 2.36 | 0.032 |
| inferiortemporal | 2.73 | 0.028 |
| isthmuscingulate | 2.39 | 0.054 |
| lateraloccipital | 2.11 | 0.028 |
| lateralorbitofrontal | 2.70 | 0.027 |
| lingual | 2.11 | 0.029 |
| medialorbitofrontal | 2.51 | 0.027 |
| middletemporal | 2.72 | 0.033 |
| parahippocampal | 2.68 | 0.055 |
| paracentral | 2.42 | 0.035 |
| parsopercularis | 2.57 | 0.035 |
| parsorbitalis | 2.72 | 0.041 |
| parstriangularis | 2.53 | 0.036 |
| pericalcarine | 1.79 | 0.027 |
| postcentral | 2.10 | 0.032 |
| posteriorcingulate | 2.53 | 0.036 |
| precentral | 2.49 | 0.043 |
| precuneus | 2.38 | 0.026 |
| rostralanteriorcingulate | 2.83 | 0.050 |
| rostralmiddlefrontal | 2.35 | 0.028 |
| superiorfrontal | 2.68 | 0.038 |
| superiorparietal | 2.19 | 0.028 |
| superiortemporal | 2.70 | 0.038 |
| supramarginal | 2.49 | 0.040 |
| frontalpole | 2.61 | 0.052 |
| temporalpole | 3.48 | 0.060 |
| transversetemporal | 2.43 | 0.047 |
| insula | 2.97 | 0.031 |

**Supplementary Table 4:** Linear regression analyses between selected regional volumes (%ICV) and Area Deprivation Indices

|  | National Percentile | | | State Decile | | |
| --- | --- | --- | --- | --- | --- | --- |
|  | β | *t* | p-value | β | *t* | p-value |
| Anterior Cingulate Cortex | -0.242 | -1.027 | 0.319 | -0.145 | -0.606 | 0.553 |
| Entorhinal Cortex | 0.103 | 0.425 | 0.676 | 0.227 | 0.96 | 0.350 |
| Fusiform Gyrus | 0.044 | 0.180 | 0.859 | 0.237 | 1.008 | 0.328 |
| Hippocampus | 0.190 | 0.819 | 0.424 | 0.129 | 0.553 | 0.587 |
| Parahippocampal Gyrus | 0.315 | -1.370 | 0.188 | **0.562** | **2.802** | **0.012*** |
| Precuneus | 0.392 | 1.758 | 0.097 | **0.609** | **3.167** | **0.006*** |
| *Motor-related regions* | | | | | | |
| Caudal Middle Frontal Gyrus | **0.457** | **2.119** | **0.049*** | **0.613** | **3.20** | **0.005*** |
| Paracentral Lobule | 0.42 | 1.920 | 0.072 | **0.51** | **2.58** | **0.019*** |
| Postcentral Gyrus | 0.36 | 1.566 | 0.136 | 0.41 | 1.85 | 0.081 |
| Precentral Gyrus | **0.55** | **2.713** | **0.015*** | **0.48** | **2.23** | **0.039*** |

***Notes significance at the 0.05 level**

**Supplementary Table 5:** Linear regression analyses between cortical thickness and Area Deprivation Indices

|  | National Percentile | | | State Decile | | |
| --- | --- | --- | --- | --- | --- | --- |
|  | β | *t* | p-value | β | *t* | p-value |
| Anterior Cingulate Cortex | 0.273 | 1.203 | 0.245 | 0.273 | 1.203 | 0.245 |
| Entorhinal Cortex | -0.145 | -0.620 | 0.543 | -0.145 | -0.620 | 0.543 |
| Fusiform Gyrus | 0.424 | 1.99 | 0.062 | 0.424 | 1.989 | 0.062 |
| Parahippocampal Gyrus | 0.381 | 1.746 | 0.098 | 0.381 | 1.746 | 0.098 |
| Precuneus | 0.197 | 0.852 | 0.406 | 0.430 | 2.023 | 0.058 |
| *Motor-related regions* | | | | | | |
| Caudal Middle Frontal Gyrus | 0.337 | 1.520 | 0.146 | **0.452** | **2.152** | **0.045*** |
| Paracentral Lobule | 0.406 | 1.883 | 0.076 | **0.574** | **2.977** | **0.008*** |
| Postcentral Gyrus | 0.354 | 1.604 | 0.126 | **0.494** | **2.409** | **0.027*** |
| Precentral Gyrus | **0.585** | **3.057** | **0.007*** | **0.577** | **3.00** | **0.008*** |

***Notes significance at the 0.05 level**

Supplementary Table 6. Top 10 correlations between NeuroTrax and regional volume measures (%ICV)

| NeuroTrax vs Regional volume | *r* |
| --- | --- |
| Visual Spatial Scores and Superior Parietal | 0.532 |
| Executive Function and Pericalcarine | 0.450 |
| Visual Spatial and Fusiform | 0.388 |
| Visual Spatial and Precuneus | 0.383 |
| Memory and Pericalcarine | 0.379 |
| Attention and Pars Opercularis | 0.374 |
| Motor Skills and Pars Opercularis | 0.361 |
| Visual Spatial and Rostral Anterior Cingulate ICV | 0.355 |
| Visual Spatial and Lateral Occipital | 0.328 |
| Global and Pars Opercularis | 0.323 |

Supplementary Table 7. Top 10 correlations between NeuroTrax and regional cortical thickness measures.

| NeuroTrax vs regional cortical thickness | *r* |
| --- | --- |
| Memory and Entorhinal | 0.623 |
| Visual Spatial and Isthmus Cingulate | 0.524 |
| Attention and Entorhinal | 0.474 |
| Executive Function and Entorhinal | 0.424 |
| Visual Spatial and Caudal Anterior Cingulate | 0.411 |
| Information Processing Speed and Entorhinal | 0.393 |
| Information Processing Speed and Frontal Pole | 0.373 |
| Memory and Fusiform | 0.341 |
| Executive Function and Frontal Pole | 0.334 |
| Executive Function and Pericalcarine | 0.327 |

Supplementary Table 8. Linear Regression Assumption of Normality, Independence, and Multicollinearity Test Statistics for Significant State Decile Findings

|  | Durbin Watson | Condition Index | Runs Test | | | Shapiro-Wilk | |
| --- | --- | --- | --- | --- | --- | --- | --- |
|  |  |  | # of runs | Z | p-value | Stat. | p-value |
| State Decile vs Global Health | 2.13 | 4.02 | 12 | 0.46 | 0.65 | .893 | .025 |
| State Decile vs Memory | 2.38 | 4.02 | 10 | 0.0 | 1 | .78 | .001 |
| State Decile vs Motor Skills | 2.56 | 4.02 | 13 | 0.28 | 0.78 | .916 | .072 |

|  | Durbin Watson | Condition Index | Runs Test | | | Shapiro-Wilk | |
| --- | --- | --- | --- | --- | --- | --- | --- |
|  |  |  | # of runs | Z | p-value | Stat. | p-value |
| State Decile vs Parahippocampal Gyrus ICV | 1.51 | 4.01 | 8 | -0.86 | 0.39 | 0.92 | 0.13 |
| State Decile vs Precuneus ICV | 1.65 | 4.01 | 10 | 0.0 | 1 | 0.98 | 0.96 |
| State Decile vs Caudal Middle Frontal Gyrus ICV | 1.47 | 4.01 | 9 | -0.46 | 0.65 | 0.98 | 0.98 |
| State Decile vs Paracentral Lobule ICV | 2.08 | 4.01 | 10 | 0.0 | 1 | 0.97 | 0.68 |
| State Decile vs Precentral Gyrus ICV | 1.94 | 4.01 | 10 | 0.0 | 1 | 0.95 | 0.44 |

|  | Durbin Watson | Condition Index | Runs Test | | | Shapiro-Wilk | |
| --- | --- | --- | --- | --- | --- | --- | --- |
|  |  |  | # of runs | Z | p-value | Stat. | p-value |
| State Decile vs Postcentral CT | 1.60 | 3.97 | 12 | 0.43 | .67 | 0.98 | 0.95 |
| State Decile vs Caudal Middle Frontal Gyrus CT | 1.26 | 3.97 | 10 | -0.23 | .82 | 0.97 | 0.87 |
| State Decile vs Paracentral Lobule CT | 1.21 | 3.97 | 6 | -2.0 | .04 | 0.97 | 0.88 |
| State Decile vs Precentral Gyrus CT | 2.30 | 3.97 | 14 | 1.4 | .16 | 0.92 | 0.08 |
